# Galloway–Mowat Syndrome Type 3 Caused by *OSGEP* Gene Variants: A Case Report and Literature Review

**DOI:** 10.3389/fped.2022.899991

**Published:** 2022-06-17

**Authors:** Suhua Xu, Lan Hu, Lin Yang, Bingbing Wu, Yun Cao, Rong Zhang, Xin Xu, Haiyan Ma, Wenhao Zhou, Guoqiang Cheng, Peng Zhang, Liyuan Hu

**Affiliations:** ^1^Department of Neonatology, National Children's Medical Center, Children's Hospital of Fudan University, Shanghai, China; ^2^Clinical Genetic Center, National Children's Medical Center, Children's Hospital of Fudan University, Shanghai, China; ^3^Shanghai Key Laboratory of Birth Defects, The Translational Medicine Center of Children Development and Disease of Fudan University, National Children's Medical Center, Children's Hospital of Fudan University, Shanghai, China; ^4^Key Laboratory of Neonatal Diseases, National Children's Medical Center, Children's Hospital of Fudan University, Shanghai, China; ^5^Department of Neonatology, Xiamen Children's Hospital, Xiamen, China; ^6^Department of Neonatology, Zhuhai Women and Children's Hospital, Zhuhai, China

**Keywords:** Galloway-Mowat syndrome, OSGEP, China Neonatal Genomes Project, nephrotic syndrome, microcephaly, case report

## Abstract

**Background:**

Galloway–Mowat syndrome type 3 (GAMOS3) is an extremely rare and severe autosomal-recessive disease characterized by early-onset nephrotic syndrome (NS), microcephaly and neurological impairment. Reported GAMOS cases have gradually increased since pathogenic *OSGEP* variants were identified as the aetiology in 2017.

**Methods:**

Using whole-exome sequencing and a data analysis process established by Children's Hospital of Fudan University, the clinical and molecular features of 3 infants with *OSGEP* mutations were summarized. Literature regarding the clinical features of GAMOS3 caused by *OSGEP* variants was reviewed.

**Results:**

Thirty-seven individuals (3 from this study) from 34 families were included. Twenty-two different *OSGEP* variants were identified. The c.740G>A (p.Arg247Gln) variant in *OSGEP* was detected in 15 families (44%), all from Asia. Most affected individuals (including patients I and II in this study) showed a typical phenotype, including microcephaly (92%) with brain anomalies (97%), developmental delay (81%), congenital NS (54%), and craniofacial (94%) and skeletal dysmorphism (84%). Renal manifestations varied from proteinuria (94%, median onset = 1.5 months) to NS (83%) and end-stage renal disease (48%, 11 months) during follow-up. Patients with congenital NS had a lower survival probability (median survival time = 3 months) than those without congenital NS (78 months) (*P* < 0.01, log-rank test).

**Conclusion:**

GAMOS3 is a progressive renal-neurological syndrome with a poor prognosis, especially with congenital NS. Microcephaly with dysmorphic features are vital clues to further evaluate renal impairment and brain anomalies. Timely molecular diagnosis is crucial for clinical decision-making, appropriate treatment and genetic counselling.

## Introduction

Galloway-Mowat syndrome (GAMOS) is a rare autosomal-recessive or X-linked recessive disorder characterized by early-onset nephrotic syndrome (NS) and microcephaly with brain anomalies and was first described in 1968 (1). Recently, pathogenic variants in the *WDR73, LAGE3, OSGEP, TP53RK, TPRKB, WDR4, NUP107, NUP133, GON7, YRDC*, and *PRDM15* genes have been identified as novel monogenic causes of GAMOS (2–8). “Galloway-Mowat syndrome 1–10 (GAMOS1–10)” is used for GAMOS with mutations in *WDR73* (OMIM 251300)*, LAGE3* (OMIM 301006)*, OSGEP* (OMIM 617729)*, TP53RK* (OMIM 617730)*, TPRKB* (OMIM 617731)*, WDR4* (OMIM 618347)*, NUP107* (OMIM 618348)*, NUP133* (OMIM 618349)*, GON7* (OMIM 619603), and *YRDC* (OMIM 619609), respectively.

As a genetically heterogeneous disease, the most prevalent genetic aetiology of GAMOS is considered to be pathogenic variants in the *OSGEP* gene (9). *OSGEP* encodes the tRNA N6-adenosine threonyl-carbamoyl-transferase protein OSGEP, a unit of the highly conserved kinase, endopeptidase and other proteins of small size (KEOPS) complex. The KEOPS complex is known to regulate a universal chemical modification of tRNAs and gene transcription, control telomere length, maintain the genome, and implicate telomere-associated DNA damage response signals (3). As a rare disease, the incidence of GAMOS is less than one in a million. Most affected individuals present with serious conditions and die in early childhood.

In this study, we present the cases of three patients diagnosed with GAMOS3. In addition, we analyse the genetic, biochemical, radiological, and clinical findings of all published GAMOS3 patients with pathogenic variants in the OSGEP gene.

## Materials and Methods

### Patient Selection and Phenotyping

Three patients who enrolled in the China Neonatal Genomes Project (CNGP) (10) and were diagnosed with GAMOS3 between August 2016 and December 2021 were included in this study. Patients' parents or legal guardians agreed and signed informed consent for DNA sequencing. Clinical information was obtained from the patients' medical records. The study was approved by the ethics committee of the Children's Hospital of Fudan University (2015-169). The patients' parents provided informed consent.

### Genetic Testing, Data Analysis, and Genetic Diagnosis

The detailed methods for our genetic analysis were previously described by Yang et al. (11). Genomic DNA was isolated from whole-blood samples, fragmented, and enriched for exome sequences using the Agilent SureSelectXT Human All Exon 50-Mb kit. The average sequencing depth of the target region of exome sequencing was 200x. Variant classifications followed the American College of Medical Genetics guidelines (12). Variants were confirmed by Sanger sequencing. The final molecular diagnosis was determined by clinicians with training in genetics based on clinical correlations between significant findings identified by exome analysis and patients' evolving phenotype (12).

### Literature Review

We conducted a thorough literature search for “Galloway–Mowat syndrome” in Medline, the China National Knowledge Infrastructure and the Wanfang Database. Publication dates ranged from January 2017 to December 2021 since pathogenic variants in the *OSGEP* gene were identified as the cause of GAMOS3 in 2017 (3, 13). Only patients with a molecular diagnosis of GAMOS3 were included, and repeated cases were excluded from the literature review. We collected the following data from the publications: the history of pregnancy and delivery, sex, gestational age at birth, birth weight, age at onset, leading signs or symptoms at onset, biochemical and imaging findings, genotype and outcomes. A total of 34 patients from 31 families, described in 6 publications (3, 9, 13–16), were included in our study.

### Statistical Analyses

The analyses were conducted using SPSS Software Version 22.0 (SPSS Inc., Chicago, IL, USA). Continuous variables are presented as medians (ranges), and categorical variables are presented as percentages. The Kaplan-Meier method was used to estimate the median survival of patients with GAMOS3, and the log-rank test was used to evaluate differences in the survival probability of congenital NS. Overall survival was defined as the time from birth to death from any cause. All statistical tests were performed with a two-sided alpha level of 0.05.

## Results

### Case Presentation

#### Patient I

The infant was born to healthy non-consanguineous parents with no significant family history. An emergency caesarean section was performed at 36 4/7 weeks due to foetal intrauterine distress. Her birth weight was 1,550 g (< third centile), and meconium-stained amniotic fluid was observed. Placental pathology showed focal infarction (1.5 cm^*^1 cm) with partial villous interstitial oedema. Neonatal resuscitation was performed immediately after birth due to the absence of spontaneous respiration and decreased heart rate. The Apgar scores at 1, 5, and 10 min were 4, 7, and 9, respectively. She was transferred to the neonatal intensive care unit (NICU) at the maternity ward for further evaluation. At 3 days of age, she developed NS with proteinuria, hypoproteinaemia with severe hypoalbuminemia, hypertriglyceridemia, hypercholesterolemia, hyponatraemia and oedema. At 6 days of age, she presented with renal impairment, more severe hypoproteinaemia, moderate ascites and neonatal necrotizing enterocolitis (NEC), despite treatment with fasting, fluid restriction and continuous albumin infusion. For further diagnosis and treatment, the patient was transferred to the NICU of our hospital at 6 days of life.

Her weight was 2,100 g (< third centile), her length was 46 cm, and her head circumference was 28 cm (< third centile) at admission. Physical examination showed generalized oedema and a variety of dysmorphic features, including coarse hair, a narrow forehead, hypertelorism, microphthalmia (left eye), blepharophimosis, sunken eyeballs, large and floppy ears, a broad nasal bridge, micrognathia, arachnodactyly, and camptodactyly.

During hospitalization, in addition to the clinical symptoms described above, the patient had hypokalaemia, hypocalcaemia, hypomagnesaemia, coagulopathy, and hypothyroidism. Abdominal ultrasound showed an indistinct renal structure and massive ascites. An electrocardiogram indicated low voltage and a prolonged Q-T interval. Echocardiography suggested patent foramen ovale. She had no neurological symptoms or signs, such as seizures and dystonia. However, multiple video electroencephalography (EEG) results suggested excessive desynchronization of electrical activity between brain hemispheres and low voltage. At 14 days of age, cranial magnetic resonance imaging (MRI) revealed periventricular leukomalacia, hypomyelination, abnormal intensity signal in the cerebral lobes, a thin cerebral cortex, gyral abnormalities, a dilation of both lateral ventricles and an enlarged extracranial space. The patient failed the hearing screening.

In view of the congenital NS and dysmorphic features, a genetic disease was suspected, and genetic testing was undertaken through trio whole-exome sequencing (WES) at 7 days of life, which identified a homozygous missense variant in the *OSGEP* gene. In addition to active correction of electrolyte disorder and acidosis, proper use of diuretics, she was commenced on daily albumin transfusions. After above treatment, the renal function of the patient gradually normalized, hypoproteinaemia improved, and ascites decreased significantly. At 41 days of age, she was discharged voluntarily and chose palliatve care. She showed a failure to thrive and finally died from cardiovascular arrest at 3 months of age.

#### Patient II

The infant was born to non-consanguineous parents with no previous history of genetic disease. A caesarean section was performed at 32 2/7 weeks due to foetal intrauterine distress. His birth weight was 1,070 g (< third centile). Oligohydramnios and a small placenta were observed. The patient's Apgar scores were 10 at both 1 and 5 min. He was hospitalized for neonatal respiratory distress syndrome and premature birth. Physical examination showed some dysmorphic features, including microcephaly, a narrow forehead, large and floppy ears, arachnodactyly and camptodactyly. He showed a developmental delay and failure to thrive. At 7 days after birth, amplitude-integrated EEG results suggested delayed background activity maturity. Cranial MRI performed at the age of 53 days revealed hypomyelination and an enlarged extracranial space.

He had persistent hypoalbuminemia from birth and was diagnosed with congenital NS with oedema and severe proteinuria at 2 months of age. He also presented hypokalaemia, hypomagnesaemia, hypothyroidism and coagulopathy. His renal function was normal during hospitalization. He showed abnormal liver function and cholestasis, which were considered to be related to cytomegalovirus infection and biliary atresia. The results of brain stem auditory evoked potential and fundus examination were normal. Trio WES were performed at 1 month of life and identified a homozygous missense variant in the *OSGEP* gene. According to the state of illness, he was given an irregular infusion of albumin, from twice a week to once a day. He was discharged voluntarily for palliative care at 3 months of age and died at the age of 5 months.

#### Patient III

The infant was born to non-consanguineous parents without a family history of hereditary diseases. He had an elder brother who was healthy. The prenatal ultrasound suggested oligohydramnios. A caesarean delivery was performed at 37 weeks. The patient's birth weight was 2,620 g, and the Apgar scores were 9 at both 1 and 5 min. He was transferred to the neonatology department because of respiratory distress. Physical examination showed microcephaly (26.5 cm, < third centile), hypertelorism and micrognathia. He had mild proteinuria at 3 days of age but had no other abnormal urinalysis results. Serum albumin, renal function parameters and renal ultrasound findings were normal. Cranial ultrasound revealed small ependymal cysts on both sides, cranial MRI failed due to unsuccessful sedation. The results of neonatal hearing screening and fundus examination were normal. At the age of 12 days, he was discharged on medical advice.

In view of the microcephaly and facial dysmorphism, trio WES was performed within first week of life and identified compound heterozygous mutations in the *OSGEP* gene. At 3 months of age, he had progressive weakness and an inability to raise his head, but refused further examinations and lost contact.

### Molecular Results

Patient I and patient II carried the same homozygous missense variant, c.740G>A p.(Arg247Gln), localized in exon 8 of the *OSGEP* gene (NM_017807), which was reported as a pathogenic mutation in the Human Gene Mutation Database. Sanger sequencing confirmed that their parents were heterozygous carriers of this *OSGEP* variant. Patient III carried a pair of compound heterozygous mutations in *OSGEP*: c.740G>A p.(Arg247Gln) was from the father, while c.560G>A p.(Gly187Val) was from the mother. The variant c.560G>A p.(Gly187Val) located in exon 6 of *OSGEP* gene was novel, which predicted as harmful variant by SIFT, Polyphen2 and MutationTaster software.

### Results of the Literature Review

#### Genetic Findings

A total of 22 different *OSGEP* (NM_017807.3) variants were identified in the 37 individuals from 34 families described, including the 3 patients who we reported (see Supplementary File 1). Twenty-three individuals had a homozygous variant, and 14 had compound heterozygous variants. Variants were categorized as missense (*n* = 18), splice site (*n* = 2), frameshift (*n* = 1), or non-sense variants (*n* = 1).

Three variants were the most common: c.740G>A; p.(Arg247Gln) was detected in 15 Asian families, 12 of which were from China; c.974G>A; p.(Arg325Gln) was detected in 5 families; and c.328T>C; p.Cys110Arg was seen in 5 families.

#### Clinical Features of Affected Individuals

In 7 of 33 (21%) families, consanguinity was reported. Fourteen individuals (40%, 14/35) had a positive family history. The most frequent clinical findings in 37 patients with GAMOS3 are summarized in Table 1. In addition, 7 patients were reported to have an electrolyte disturbance, especially hypomagnesaemia (86%, 6/7), and 4 patients had visual and/or hearing impairment (see Supplementary Table 1).

**Table 1:** Clinical features of 37 patients with Galloway-Mowat syndrome type 3.

**Finding**	**Cohort, *n =* 3**	**Literature, *n =* 34**	**Total, *n =* 37**	**Median Age of Onset (Range)**
**Male**	2/3	20/34	22/37 (59%)	
**Preterm birth**	2/3	5/18	7/21 (33%)	
**Abnormal prenatal findings**	3/3	13/15	16/18 (89%)	
IUGR	2/3	10/15	12/18 (67%)	
Oligohydramnios	2/3	7/15	9/18 (50%)	
Microcephaly	0/3	6/15	6/18 (33%)	
**Neurological involvement**	3/3	33/34	36/37 (97%)	
Microcephaly	3/3	31/34	34/37 (92%)	
Developmental delay	3/3	27/34	30/37 (81%)	
Hypotonia	2/3	17/34	19/37 (51%)	
Seizures	0/3	16/34	16/37 (43%)	
Failure to thrive	2/3	5/34	7/37 (21%)	
Spasticity	0/3	5/34	5/37 (14%)	
**Brain anomalies**	2/2	29/30	31/32 (97%)	
Gyral abnormalities	1/2	17/31	18/33 (55%)	
Myelinationdefects/white matter anomalies	2/2	15/31	17/33 (52%)	
Brian atropy	0/2	15/31	15/33 (45%)	
**Renal phenotype**
Proteinuria	3/3	31/33	34/36 (94%)	1.5 m (0–13 y)
NS	2/2	27/33	29/35 (83%)	
Congenital NS	2/3	17/32	19/35 (54%)	
ESRD	0/1	12/24	12/25 (48%)	11 m (1 m−12.5 y)
**Renal biopsy**	0	13	13	
FSGS	0	7/13	7/13 (54%)	
DMS	0	3/13	3/13 (23%)	
Foot process effacement	0	2/13	2/13 (15%)	
**Facial dysmorphism**	3/3	26/28	29/31 (94%)	
**Skeletal deformity**	2/3	24/28	26/31 (84%)	

*DMS, diffuse mesangial sclerosis; ESRD, end stage renal disease; FSGS, focal segmental glomerulosclerosis; M, month; NS, nephrotic syndrome; IUGR, intrauterine growth retardation; Y, year*.

**Figure 1:**
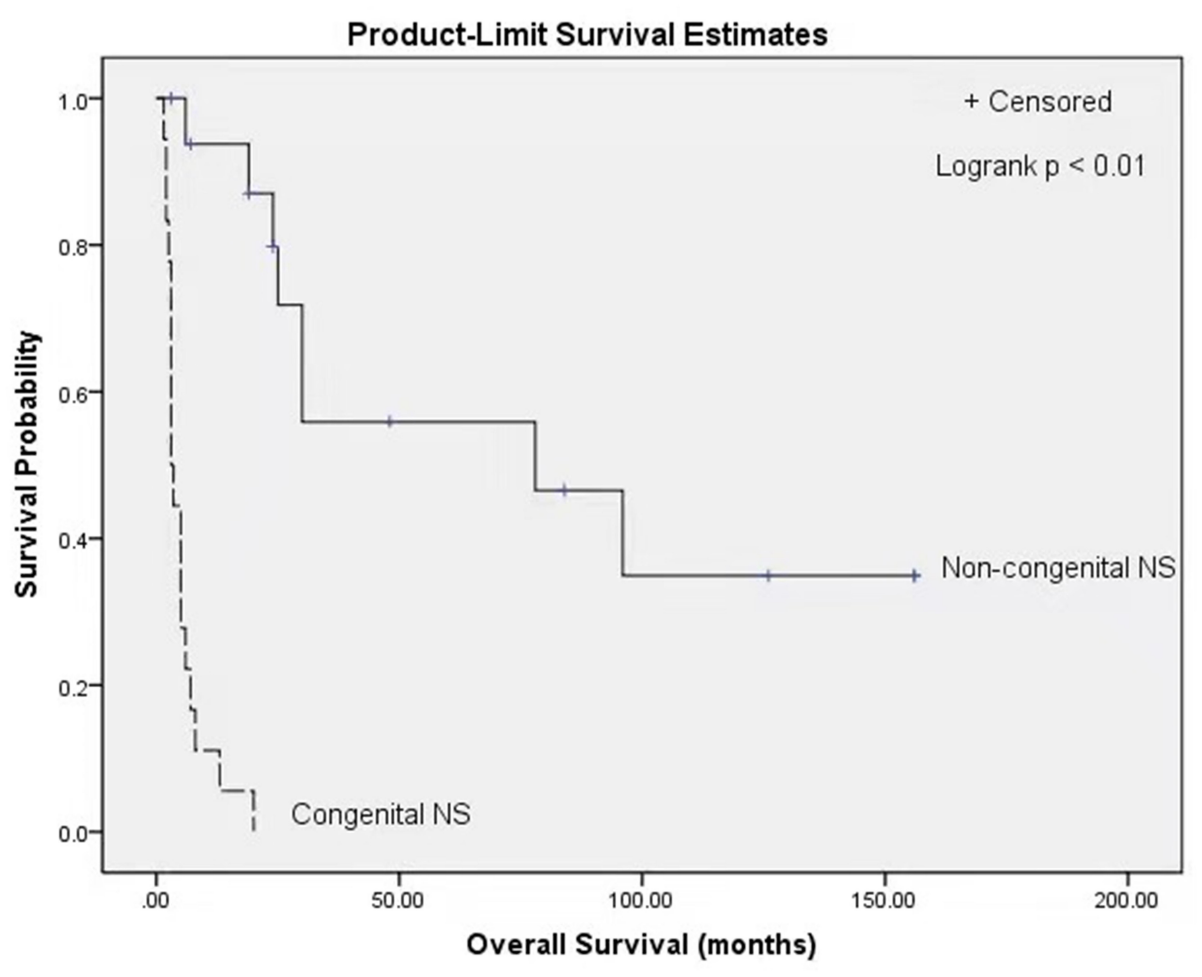
Kaplan Meier survival curves stratified by classification of congenital NS (congenital NS: *n* = 18, non-congenital NS: *n* = 17; *p* < 0.01, log-rank test). NS, Nephrotic syndrome.

At the end of follow-up in each of the case reports, 9 of 36 patients were alive (25%), and 27 had died (75%). The median survival for the population was 8 months. Patients with congenital NS showed a median survival of 3 months and had a lower probability of survival than patients without congenital NS, who had a median survival of 78 months (*P* < 0.01, log-rank test) (Figure 1).

The causes of death included unknown cause (59%, 16/27), multiorgan failure (22%, 6/27), cardiopulmonary collapse (11%, 3/27), hypertensive crisis (4%, 1/27) and neurological deterioration (4%, 1/27).

## Discussion

We reported the cases of 3 unrelated patients with GAMOS3, 2 of whom presented with typical renal-neurological manifestations, including congenital NS, microcephaly, developmental delay and early death. The remaining patient primarily manifested microcephaly and hypotonia with no severe renal phenotype during the follow-up period. Combining these three patients with the findings of a literature review, we summarized the phenotypes and genotypes of the 37 patients with GAMOS3 from 34 families who had a definitive molecular diagnosis.

We found a total of 22 different *OSGEP* sequence variants which was located throughout the whole gene. More missense variants than loss-of-function (splice site, frameshift, and stope) variants were found, with a respective ratio of 18:4, and none of the affected patients carried two truncating alleles in the mutated gene. The c.740G>A; p.(Arg247Gln) variant was detected in 44% (15/34) of families, all of which came from Asia, especially China (35%, 12/34), indicating that this variant could be a founder variant in the Asian population. No correlation between phenotype and genotype was apparent. Braun et al. used a yeast-based functional growth complementation to identify two functional classes of *OSGEP* mutant alleles: hypomorphic alleles (Lys198Arg, Arg247Gln, Arg280Cys, and Arg325Gln), which restore growth but to a lesser extent than the wild-type *OSGEP*, causing a mild phenotype, and amorphic alleles (Ile14Phe, Ile111Thr, and Cys110Arg), which completely fail to restore growth, leading to a severe phenotype (3). Although three patients carrying homozygous c.974G>A (p.Arg325Gln) in *OSGEP* survived longer than 6 years (3, 13, 17), all patients with homozygous c.740G>A (p.Arg247Gln) in *OSGEP* developed severe encephalopathy and died before 6 months of age (3, 15, 16).

The clinical phenotype of the 37 individuals was homogenous in terms of the specific clinical findings reported, including early- or late-onset NS, microcephaly with gyral abnormalities, developmental delay, seizure, hypotonia, and craniofacial and skeletal dysmorphism (3, 9). The prognosis for patients with GAMOS3 was poor, with a median survival of 8 months. Microcephaly (92%, 34/37) seems to be the more important clue for the timely diagnosis of GAMOS3 than early-onset NS. Braun et al. found that the knockout of the *OSGEP* gene in zebrafish and mice resulted in microcephaly without a renal phenotype, possibly due to early lethality in animal models (3). Microcephaly (33%, 6/18) in the second or third trimester has been detected by prenatal ultrasonography in patients with GAMOS3, and gyral and myelin abnormalities have been identified in 2 patients by foetal MRI (16). In addition, 22% (8/37) of patients presented with predominantly neurological involvement, especially microcephaly, and had not developed NS before death/report. In our patients, the microcephaly observed in patient III was the main indicator for the timely diagnosis. This finding implies that neurological abnormalities are prevalent in GAMOS3, often prior to a renal phenotype, and even in the prenatal stage. We suggest that microcephaly should be considered an important factor in the diagnosis of GAMOS3. For patients in whom GAMOS 3 is suspected, foetal MRI and postpartum cranial MRI are useful tools to detect typical cranial imaging findings, including gyral abnormalities, myelination defects, brain atrophy, and other brain anomalies, such as corpus callosum dysplasia.

Renal phenotypic variability has been described in GAMOS3, ranging from different degrees of proteinuria (94%, 34/36), with a median age at onset of 1.5 months (ranging from birth to 13 years), to NS (83%, 29/35) and end-stage renal disease (48%, 12/25), with a median age at onset of 11 months (ranging from 1 month to 12.5 years) during the follow-up period. Fifty-four percent (19/35) of patients rapidly developed NS before 3 months of age, including our patients I and II. Moreover, we found that the median survival time (3 months) of patients with congenital NS was significantly lower than that of patients without congenital NS (78 months), suggesting that patients with congenial NS have an overall lower probability of survival. Braun et al. demonstrated that the knockdown of *OSGEP* and *TP53RK* in human podocytes caused defects in the actin cytoskeleton and decreased the podocyte migration rate (3), which could contribute to the development of NS. Renal biopsy revealed that the main glomerular light microscopy findings were focal segmental glomerulosclerosis (54%, 7/13) and diffuse mesangial sclerosis (23%, 3/13). Electron microscopy revealed an irregular thickening of the glomerular basement membrane with or without diffuse podocyte foot process effacement. Since GAMOS3 is not an immune disease, congenital NS in GAMOS3 is theoretically almost always resistant to glucocorticoids and immunosuppressants. Currently, no effective treatment for congenital NS in GAMOS3 is available. In our clinical experience, appropriate supportive care, including the maintenance of internal homeostasis, adequate nutrition and the prevention and treatment of infection (18), may temporarily alleviate clinical symptoms.

Renal damage caused by GAMOS3 involves not only the glomerulus but also the tubules. Three patients who had identical homozygous mutations (c.974G>A, p.Arg325Gln) demonstrated tubulopathy, including tubular proteinuria, hypermagnesuria with hypercalciuria, and hypomagnesaemia. Hypomagnesaemia with or without hypocalcaemia was reported in 86% (6/7) of GAMOS3 patients with electrolyte disorders. The KEOPS complex has been shown to play a significant role in mitochondrial function (19), and mitochondrial dysfunction has been linked to renal alterations, including tubular dysfunction and hypomagnesaemia (20). In consideration of the various renal manifestations of OSGEP mutations, patients should undergo a complete nephrological workup.

One of the features of GAMOS is an “aged face,” including large and floppy ears, micrognathia, hypertelorism, microphthalmia, a narrow or receding forehead, and prominent glabella with a broad nasal bridge, as in patients I and II. Lin et al. suggested that a careful evaluation of facial features can provide useful clues for an early and accurate diagnosis of GAMOS3 (16). According to the literature review, 94% (29/31) of individuals had facial dysmorphism, although some patients did not present the above peculiar facial dysmorphism or even lacked facial description information. With a better understanding of GAMOS3, the dysmorphic features of the patients reported recently have been mentioned in more detail. Arachnodactyly and camptodactyly have also been frequently observed in patients with GAMOS3. Other skeletal abnormalities, such as clenched hands, flexion contracture of joints, dislocated hips, talipes calcaneovalgus, have also been reported (3, 9, 13–16).

## Conclusion

In summary, we presented the cases of 3 patients with GAMOS3 caused by *OSGEP* pathogenic variants. The genotype and phenotype of GAMOS3 were summarized by reviewing the literature in the past 5 years. The c.740G>A (p.Arg247Gln) variant in *OSGEP* seems to be a founder variant in the Asian population that carries this homozygous variant, resulting in a severe disease phenotype and an extremely poor prognosis. GAMOS3 should be suspected when patients have microcephaly with special facial features, which indicates the need to examine whether proteinuria and brain anomalies are present to provide more diagnostic clues. Patients with congenital NS have a lower median survival time than those without congenital NS. Timely molecular diagnosis through gene sequencing is crucial for clinical decision-making and genetic counselling.

## Data Availability Statement

The datasets for this article are not publicly available due to concerns regarding participant/patient anonymity. Requests to access the datasets should be directed to the corresponding author.

## Ethics Statement

The studies involving human participants were reviewed and approved by the Research Ethics Committee of the Children's Hospital of Fudan University (2015-169). Written informed consent to participate in this study was provided by the participants' legal guardian/next of kin.

## Author Contributions

GC, LiH, PZ, and WZ contributed to conception and design of the study. LY and BW performed the genetic experiments and data analysis. LiH, SX, and LaH collected the data. SX wrote the first draft of the manuscript. All authors contributed to clinical care of patients, manuscript revision, read, and approved the submitted version.

## Funding

This research was supported by Shanghai Municipal Science and Technology Major Project, Grant/Award Number: 2018SHZDZX01 and 2018SHZDZX05.

## Conflict of Interest

The authors declare that the research was conducted in the absence of any commercial or financial relationships that could be construed as a potential conflict of interest.

## Publisher's Note

All claims expressed in this article are solely those of the authors and do not necessarily represent those of their affiliated organizations, or those of the publisher, the editors and the reviewers. Any product that may be evaluated in this article, or claim that may be made by its manufacturer, is not guaranteed or endorsed by the publisher.
