# Supplementary material for: Galloway–Mowat Syndrome Type 3 Caused by OSGEP Gene Variants: A Case Report and Literature Review
Source: Front Pediatr. 2022 Jun 17;10:899991. doi: 10.3389/fped.2022.899991 (PMC9249162; doi:10.3389/fped.2022.899991)
Supplement: Supplementary file 1 [file Data_Sheet_1.PDF]

Supplementary Table 1 The clinical manifestations and gene mutations of 37 patients with Galloway-Mowat syndrome type 3

Case no.	Nucleotide change (Amino acid change)	Exon (Zygosity)	Gender/GA (w)/BW (g)/Ethnic origin	Parental Consanguinity/Family medical history/Prenatal findings	Proteinuria (onset)/NS/ESRD (onset)/Renal biopsy (at age)	Brain manifestations	Cranial MRI findings	Dysmorphic features	Other manifestations	Outcome
1[9]	c.81C>G (p.Asn27Lys)	Exon 1 (Hom)	M/39+3/3400/Spanish	Y/N/NA	Y (1.5m)/congenital NS/Y/DMS, tubular atrophy, primitive glomeruli	microcephaly, hypotonia, developmental delay	brain atrophy, poor myelination	no	hyperkalemia, hyponatremia, hypomagnesemia	died of progressive neurological deterioration at 8m
2[3]	c.1A>T; c.839G>T (p.Arg280Leu)	Exon 1 (het); Exon 9 (het)	M/NA/NA/Caucasian, Hispanic	N/N/NA	Y (14m)/NS/NA/FSGS (15m)	microcephaly, myoclonic seizures, developmental delay, hypotonia, spasticity	enlarged subdural/subarachnoidal spaces, poor myelination, gyral simplification, cerebellar atrophy	large and floppy ears, pinched nose	visual impairment	dead at 19m
3[3]	c.40A>T (p.Ile14Phe)	Exon 1 (Hom)	F/preterm/NA/Iran, Caucasian	Y/previous child of the family had microcephaly, seizures, renal failure (at 7m) and died at 13m due to status epilepticus/NA	Y (4m)/infantile SRNS/Y (2y)/NA	microcephaly, globle developmental delay, spasticity, seizures	NA	large and floppy ears, short stature	ASD	dead at 30m
4[3]	c.40A>T (p.Ile14Phe)	Exon 1 (Hom)	M/NA/NA/Iran, White, Persian, Kurdish	Y/two siblings of mother died neonatally of unknown cause/NA	Y (5m)/SRNS/Y (22m)/FSGS (10m)	microcephaly, abnormalities in motor development, speech delay, spasticity	NA	strabism, short stature	N	dead at 25m
5[9]	c.157A>T (p.Ile53Phe)	Exon 2 (Hom)	F/40+2/2940/Pakistani	Y/N/NA	Y (2.5m)/congenital NS/NA/increased glomerular mesangial matrix	microcephaly, hypotonia, developmental delay, seizures, failure to thrive	brain atrophy	wide nasal bridge, aquiline nose, retrognathia, low set ears, arachnodactyly	electrolyte disorders	died of cardiorespiratory arrest in a sepsis context at 7m
6[3]	c.232A>G (p.Lys78Glu); c.973C>T (p.Arg325Trp)	Exon2 (het); Exon11 (het)	M/NA/NA/Hispanic	N/N/NA	Y (6m)/infantile NS/Y (24m)/FSGS (6m)	microcephaly, hypotonia, hyperreflexia, developmental delay, speech delay	poor myelination	facial dysmorphism, esotropia, camptodactyly	PDA, ASD	alive with ESRD under RRT at 24m
7[15]	c.133dupA; c.608C>T (p.Ser203Leu)	Exon 2 (het); Exon 6 (het)	F/39+6/3100/China	N/previous child of the family had microcephaly, seizures, global developmental delay, hydrocephalus, facial dysmorphism (esotropia, low nasal bridge and long philtrum), and died at 8y/(US) a small biparietal diameter	Y (4y)/N/N/N	microcephaly, hypotonia, global developmental delay, failure to thrive	brain atrophy	esotropia, deeply-set eye, low nasal bridge, long philtrum, single transverse palmar crease, calcaneovalgus deformity	N	alive at 4y
8[3]	c.319G>A (p.Val107Met)	Exon 3 (Hom)	M/NA/NA/European	N/parental second cousin with ESRD during adolescence/NA	Y (0.5m)/congenital NS/Y (2.5m)/NA	microcephaly, hypotonia, developmental delay, seizures	pachygria, polymicrogyria	arachnodactyly, camptodactyly	N	dead at 2.5m
9[3]	c.328T>C (p.Cys110Arg); c.332T>C (p.Ile111Thr)	Exon 3 (het); Exon 3 (het)	F/NA/NA/Netherlands	N/N/oligohydramnios	Y (1m)/congenital NS/Y (1m)/DMS	microcephaly, developmental delay	reduced gyration, cerebellar hypoplasia, abnormal myelination	long convex beaked nose, thin upper lip, down turned corners of the mouth, micrognathia, pointed chin, arachnodactyly, adducted thumbs, dislocated hips, talipes calcaneovalgus	N	dead at 1.5m
10[3]	c.328T>C (p.Cys110Arg); c.530G>C (p.Gly177Ala)	Exon 3 (het); Exon 5 (het)	F/NA/NA/USA	N/N/NA	N/N/NA/NA	microcephaly, seizures	lissencephaly, hypogenesis of corpus callosum	large and floppy ears, unusual fat distribution, congenital hip dislocation	poor wound healing	dead at 2.5y
11[3]	c.328T>C (p.Cys110Arg); c.530G>C (p.Gly177Ala)	Exon 3 (het); Exon 5 (het)	M/NA/NA/USA	N/N/NA	N/N/NA/NA	microcephaly	lissencephaly	large and floppy ears, unusual fat distribution	poor wound healing	alive at 7m
12[3]	c.328T>C (p.Cys110Arg); c.838C>T (p.Arg280Cys)	Exon 3 (het); Exon 9 (het)	F/NA/NA/USA	N/N/NA	Y (19m)/NS/NA/FSGS	microcephaly, seizures, developmental delay, speech delay, repetitive movements	cerebellar atrophy	NA	N	alive at 19m
13*[3]	c.328T>C (p.Cys110Arg); c.974G>A (p.Arg325Gln)	Exon 3 (het); Exon11 (het)	M/NA/NA/Caucasian	N/affected siblings/NA	Y (3m)/congenital NS/Y (11m)/NA	microcephaly, myoclonus, developmental delay	lissencephaly	narrow forehead, large, low-set ears, small mouth, micrognathia, short stature	hiatal hernia	dead at 13m
14*[3]	c.328T>C (p.Cys110Arg); c.974G>A (p.Arg325Gln)	Exon 3 (het); Exon11 (het)	M/NA/NA/Caucasian	N/affected siblings/NA	Y (at birth)/congenital NS/NA/NA	microcephaly, seizures, spasticity, developmental delay	NA	NA	N	NA
15[3]	c.416T>C (p.Ile139Thr)	Exon 4 (Hom)	M/NA/NA/Turkish	Y/N/NA	Y (13y)/SRNS/N/FSGS	no	NA	NA	diabetes mellitus type II	alive at 13y
16[3]	c.416T>C (p.Ile139Thr)	Exon 4 (Hom)	F/NA/NA/Turkish	Y/N/NA	Y (11m)/SRNS/Y (12.5y)/FSGS	recurrent headaches, double vision	retro bulbar intra orbital lymphatic malformation	NA	aneurysm of the ascending aorta	alive at 13y
17[3]	c.593A>G (p.Lys198Arg); c.740G>A (p.Arg247Gln)	Exon 6 (het); Exon 8 (het)	F/NA/NA/Asia	N/N/NA	Y (14m)/infantile NS/NA/NA	microcephaly, seizures, intellectual disability, delay motor milestones, hypotonia	brain atrophy, atrophic corpus callosum, smaller ventral pons, enlarged subdural/subarachnoid spaces	ptosis, entropion repair, short stature	NA	dead at 2y
18*[3]	c.740G>A (p.Arg247Gln)	Exon 8 (Hom)	M/37/2118/China	N/affected siblings/(US at 32w) oligohydramnions, microcephaly; (fetal MRI at 34w) pachygyria, myelination defects; IUGR	Y (1m)/congenital NS/Y (4m)/NA	microcephaly, seizures, developmental delay, hypotonia	pachygyria, poor myelination	large and floppy ears, micrognathia, hypertelorism, microphthalmia, sunken eyeballs, coarse hair, a narrow forehead, a beak nose, prominent glabella with a broad nasal bridge, high arch palate, camptodactyly, arachnodactyly, clenched hands, mild hip contractures	cryptorchidism, micropenis, ectopic kidney, auditory and visual dysfunction	died of multiorgan failure at 5m
19*[3]	c.740G>A (p.Arg247Gln)	Exon 8 (Hom)	F/40/2496/China	N/affected siblings/(US at 27w) oligohydramnions, microcephaly; (fetal MRI at 32w) pachygyria, cerebellar atrophy; IUGR	Y (2d)/congenital NS/N/NA	microcephaly, seizures, developmental delay, hypotonia	pachygyria, poor myelination, cerebellar atrophy	large and floppy ears, micrognathia, hypertelorism, microphthalmia, sunken eyeballs, a narrow forehead, a beak nose, prominent glabella with a broad nasal bridge, high arch palate, arachnodactyly	larygomalacia, swallowing disturbance, significant lactic aciduria	died of multiorgan failure at 3m
20[3]	c.740G>A (p.Arg247Gln)	Exon 8 (Hom)	M/40/2496/China	N/affected siblings/oligohydramnios, IUGR	Y (at birth)/congenital NS/Y (1m)/NA	microcephaly, developmental delay, opisthotonos	broad gyri and hypoplasia of sulci in frontotemporal areas, possible leukoencephalopathy, subdural fluid collection	hypertelorism, deep-set eyes, micrognathia, arachnodactyly	lactic aciduria, pulmonary edema	dead at 3m
21[3]	c.740G>A (p.Arg247Gln)	Exon 8 (Hom)	M/36/2345/China	N/N/(US at 34w) oligohydramnions, microcephaly, IUGR	Y (at birth)/congenital NS/N/NA	microcephaly, hypotonia, developmental delay	pachygyria, hypomyelination	large and floppy ears, micrognathia, hypertelorism, microphthalmia, sunken eyeballs, a narrow forehead, a beak nose, prominent glabella with a broad nasal bridge, high arch palate, arachnodactyly	micropenis, mild hearing impairment	died of multiorgan failure at 3m
22[3]	c.740G>A (p.Arg247Gln)	Exon 8 (Hom)	F/39/2460/China	N/N/IUGR, microcephaly	Y (1.5m)/congenital NS/N/light microscopy-mild glomerular changes, cystic dilatation of tubular lumina, tubular atrophy with proteinaceous casts, and arteriolar medial hypertrophy; electron microscopy-an irregular thickness of the glomerular basement membrane, complete effacement of the foot processes	microcephaly, seizures, hypotonia, developmental delay	pachygyria, hypomyelination	large and floppy ears, micrognathia, hypertelorism, microphthalmia, sunken eyeballs, a narrow forehead, a beak nose, prominent glabella with a broad nasal bridge, high arch palate, arachnodactyly	hearing and visual dysfunction	died of multiorgan failure at 3m
23[3]	c.740G>A (p.Arg247Gln)	Exon 8 (Hom)	M/36/2350/China	N/N/IUGR	Y (at birth)/congenital NS/N/NA	microcephaly	simplified gyri and sulci, pachygyria in the frontal lobes, reduced density of the frontal white matter	micrognathia, arachnodactyly	N	dead at 3.5m
24[3]	c.740G>A (p.Arg247Gln)	Exon 8 (Hom)	M/28/840/China	N/N/IUGR	Y (1m)/congenital NS/N/NA	microcephaly	brain atrophy, hypodense cerebral white matter, thin corpus callosum	floppy ears, micrognathia, arachnodactyly	N	dead at 6m
25[3]	c.740G>A (p.Arg247Gln)	Exon 8 (Hom)	M/39/2034/China	N/N/IUGR	Y (1m)/congenital NS/N/diffuse foot process effacement	microcephaly	cerebral and cerebellar atrophy, simplified frontal and temporal gyration, white matter changes	flat nasal bridge, micrognathia, arachnodactyly	abnormal EEG (diffuse cortical dysfunction)	dead at 5m
26[3]	c.740G>A (p.Arg247Gln)	Exon 8 (Hom)	F/34/NA/Asia	N/N/NA	Y (at birth)/congenital NS/NA/collapsing FSGS	microcephaly, hypotonia	cerebellar vermis atrophy with prominent interfoliate sulci, thrombosed left transverse sinus, underdeveloped cortical ribbon	hypertelorism, down slanting palpebral fissures, camptodactyly	ASD	dead at 2m
27[17]	c.740G>A (p.Arg247Gln)	Exon 8 (Hom)	M/38/2340/China	N/NA/(US at 35w) oligohydramnions, microcephaly, IUGR	Y (1m)/congenital NS/NA/light microscopy-DMS with increase of mesangial cells and matrix; electron microscopy-global multilayering and irregular thickening of glomerular basement membrane	microcephaly, hypotonia, developmental delay, seizures	pachygyria (CT)	large and floppy ears, micrognathia, hypertelorism, microphthalmia, sunken eyeballs, coarse hair, a narrow forehead, a beak nose, prominent glabella with a broad nasal bridge, high arch palate, arachnodactyly, camptodactyly	micropenis	died of multiorgan failure at 2m
28 (Patient I)	c.740G>A (p.Arg247Gln)	Exon 8 (Hom)	F/36+4/1500/Asia	N/N/IUGR	Y (2d)/congenital NS/N/N	microcephaly, developmental delay, hypotonia, faliure to thrive	periventricular leukomalacia, hypomyelination, gyral abnormalities, bilateral lateral ventricle dilation	narrow forehead, hypertelorism, microphthalmia, large and floppy ears, broad nasal bridge, micrognathia, arachnodactyly, camptodactyly	neonatal asphyxia, renal impairment, ascites, electrolyte disturbance(hyponatremia, hypokalemia, hypocalcemia, hypomagnesemia), marked abnormal EEG	died of cardiorespiratory arrest at 3m
29 (Patient II)	c.740G>A (p.Arg247Gln)	Exon 8 (Hom)	M/32+2/1070/China	N/N/IUGR, oligohydramnions	Y (2m)/congenital NS/NA/N	microcephaly, developmental delay, faliure to thrive	hypomyelination, enlarged extracranial space	narrow forehead, large and floppy ears, arachnodactyly, camptodactyly	cytomegalovirus infection, biliary atresia, electrolyte disturbance, abnormal EEG	dead at 5m
30[16]	c.740G>A (p.Arg247Gln)	Exon 8 (Hom)	M/NA/NA/China	NA/NA/NA	NA/NA/NA/NA	focal refractory seizures, developmental delay	lissencephaly	NA	NA	dead at 4m
31[3]	c.740G>A (p.Arg247Gln); c.411+12_411+22delins9	Exon 8 (het); Intron 3 (het)	F/38/1954/China	N/previous child of the family had renal failure from age 5m and died at 13m/(US at 38w) oligohydramnions, microcephaly, IUGR	Y (2d)/congenital NS/Y (19m)/NA	microcephaly, hypotonia, developmental delay, seizures	pachygyria (CT)	large and floppy ears, micrognathia, hypertelorism, microphthalmia, sunken eyeballs, a narrow forehead, a beak nose, prominent glabella with a broad nasal bridge, arachnodactyly	N	died of multiorgan failure at 20m
32 (Patient III)	c.740G>A (p.Arg247Gln); c.560G>A (p.Gly187Val)	Exon 8 (het); Exon 6 (het)	M/37/2620/China	N/N/oligohydramnions	Y (3d)/NA/NA/NA	microcephaly, developmental delay, hypotonia	small ependymal cysts on both sides (US)	hypertelorism, micrognathia	N	alive at 3m
33[3]	c.974G>A (p.Arg325Gln); c.236-2delA	Exon 11 (het); Exon 3 (het)	M/NA/NA/Jordan	N/sister affected and deceased/NA	Y (6m)/infantile NS/Y (6m)/NA	microcephaly, developmental delay	cerebral atrophy, bilateral lissencephaly predominantly in frontotemporal regions, thin corpus callosum	low-set ears, hypertelorism, small down ward slanting eyes	N	dead at 6m
34[3]	c.974G>A (p.Arg325Gln); c.839G>A (p.Arg280His)	Exon 11 (het); Exon 9 (het)	M/NA/NA/Africa	N/older brother affected (SRNS at 22m) and deceased from ESKD at 14y/NA	Y (13m)/NS/N/NA	microcephaly, developmental delay, aggressive behavior	myelination delay, cerebellar atrophy, atrophy of upper spinal cord and medulla	NA	N	alive at 10.5y
35*[14]	c.974G>A (p.Arg325Gln)	Exon 11 (Hom)	F/term/2600/Arab	Y/affected siblings/normal	Y/N/N/N	microcephaly, hypotonia, global developmental delay, failure to thrive	cerebellar atrophy	microphthalmia, nystagmus,upturned nose, antimongoloid slant, high-arched palate, thoracolumbar kyphosis	hypertention, hypomagnesemia	died of hypertensive crisis at 6.5y
36*[14]	c.974G>A (p.Arg325Gln)	Exon 11 (Hom)	M/term/3800/Arab	Y/affected siblings/a hypoplastic left heart and aortic coarctation	Y/N/N/N	microcephaly, nystagmus, hypotonia, global developmental delay, failure to thrive	leukodystrophy	frontal bossing, microphthalmia, bilateral coloboma, bilateral ptosis, antimongoloid slant, strabismus, nystagmus, pectus excavatum and chest wall asymmetry, arachnodactyly	a hypoplastic left heart, aortic coarctation, cryptorchidism, hypomagnesemia, hypocalcemia	died of respiratory insufficiency at 8y
37[3, 18]	c.974G>A (p.Arg325Gln)	Exon 11 (Hom)	F/term/3288/Caucasian (American Indian)	N/N/normal	Y (3.5y)/N/N/N	microcephaly, globle developmental delay, hypotonia (trunk), spasticity (lower limbs), status epilepticus, failure to thrive	hypomyelination, atrophy of the cerebellum and the brain stem	large ears, frontal bossing, broad nasal bridge, high-arched palate, scarce eyebrows	recurrent urinary tract infections, neurogenic bladder dysfunction, partial Fanconi syndrome,bladder stone and nephrolithiasis,	alive at 7y

ASD, atrial septal defect; BW, birth weight; CT, computerized tomography; DMS, diffuse mesangial sclerosis; d, day; EEG, electroencephalography; ESRD, end stage renal disease; FSGS, focal segmental glomerulosclerosis; F, female; GA, gestational age; het, heterozygous; hom, homozygous; IUGR, intrauterine growth retardation; M, male; N, no; NA, not available; NS, nephrotic syndrome; m, month; MRI, magnetic resonance imaging; PDA, patent ductus arteriosus; RRT, renal replacement therapy; SRNS, steroid resistant nephrotic syndrome; USA, United States Of America; US, ultrasonography; Y, yes; y, year; w, week.; * = individuals from one family.
